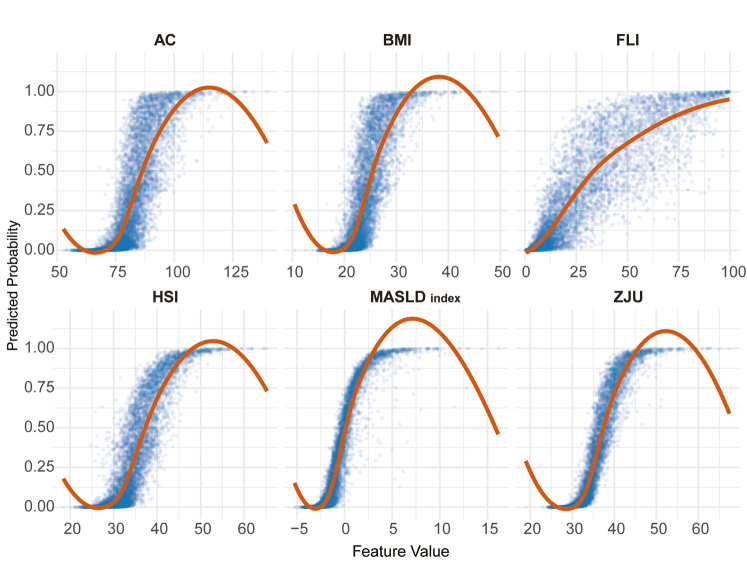# Development of a Neural Network to Detect Hepatic Steatosis in Metabolic Dysfunction–Associated Steatotic Liver Disease

**DOI:** 10.1016/j.gastha.2025.100765

**Published:** 2025-08-19

**Authors:** Masashi Hirooka, Teruki Miyake, Ryo Yano, Yoshiko Nakamura, Yuki Okazaki, Toyoki Shimamoto, Yasunori Yamamoto, Takao Watanabe, Osamu Yoshida, Kana Hirooka, Yoshio Tokumoto, Masanori Abe, Takeru Iwata, Yoichi Hiasa

**Affiliations:** 1 Total Medical Support Center, Ehime University Hospital, Toon, Japan; 2 Department of Gastroenterology and Metabology, Ehime University Graduate School of Medicine, Toon, Japan; 3 Department of Gastroenterology and Metabology, National Hospital Organization Ehime Medical Center, Toon, Japan; 4 Ehime General Health Care Association, Matsuyama, Japan

**Keywords:** artificial intelligence, hepatic steatosis, metabolic dysfunction, neural network model, noninvasive screening

## Abstract

**Background and Aims:**

Early identification of metabolic dysfunction–associated steatotic liver disease (MASLD) is critical for risk stratification and timely intervention. Conventional noninvasive indices (fatty liver index, hepatic steatosis index, Zhejiang University index, and MASLD index) are limited by linear assumptions and moderate predictive accuracy. We aimed to develop and externally validate a neural network model for noninvasive detection of hepatic steatosis.

**Methods:**

We used a retrospective cohort of 17,465 Japanese health checkup participants (2010–2020). All clinical data were obtained at the Ehime General Health Care Association. The cohort was split into a development cohort (n = 8426) and an internal validation cohort (n = 9039). A feedforward neural network was trained using clinical and biochemical variables, including body mass index, abdominal circumference, and existing indices. External validation used the Third National Health and Nutrition Examination Survey cohort (n = 9759), with hepatic steatosis defined by ultrasonography (Gallbladder and Upper Abdominal Ultrasound Hepatic Steatosis Profile Rating≥2). Model performance was assessed via area under the receiver operating characteristic curve, calibration, decision curve analysis, and subgroup analyses.

**Results:**

The neural network achieved an area under the receiver operating characteristic curve of 0.922 (95% confidence interval: 0.913–0.931) in internal validation and 0.924 (95% confidence interval: 0.917–0.931) in external validation, outperforming fatty liver index, hepatic steatosis index, Zhejiang University index, and MASLD indices (all *P* < .001). At the optimal Youden cutoff (0.357), sensitivity and specificity were 89% and 86%, respectively. Calibration analysis and decision curve analysis confirmed strong agreement between predicted and observed risk. Abdominal circumference and body mass index were the most influential predictors.

**Conclusion:**

Our neural network model outperforms conventional indices in detecting moderate-to-severe hepatic steatosis and may facilitate early, scalable MASLD screening in primary care and low-resource settings.

UMIN No. 11953.

## Graphical abstract

**Figure:**
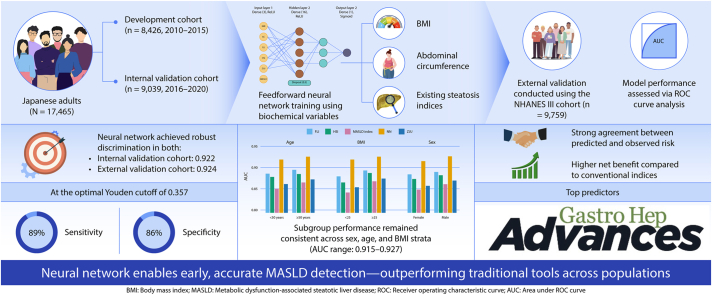

## Introduction

Metabolic dysfunction–associated steatotic liver disease (MASLD) is now recognized as the most prevalent chronic liver disease worldwide, with a global prevalence estimated at 32%.^1^^,^^2^ MASLD is closely linked not only to metabolic syndrome but also to adverse outcomes such as cardiovascular disease, chronic kidney disease, diabetes, malignancies, and liver-related mortality.^3^^,^^4^

Recent multisociety guidelines, including those by the European Association for the Study of the Liver,^5^ American Association for the Study of Liver Diseases,^6^ and the Japan Society of Hepatology,^7^ emphasize a stepwise diagnostic algorithm for MASLD that begins with detecting hepatic steatosis, followed by fibrosis risk stratification. In this framework, ultrasound B-mode imaging is considered the primary tool for identifying hepatic steatosis. However, many health-care settings lack access to skilled operators or sufficient imaging infrastructure, limiting effective implementation of this strategy.^8^

To overcome such barriers, several noninvasive indices, such as the Fatty Liver Index (FLI),^9^ Hepatic Steatosis Index (HSI),^10^ and Zhejiang University (ZJU) index^11^—have been proposed to screen for steatosis based on routine clinical parameters. More recently, the MASLD index, initially known as the nonalcoholic fatty liver disease index at the time of its development, has gained attention for its utility in identifying individuals with metabolic dysfunction–associated liver abnormalities.^12^ Despite their practicality, these indices often underperform across diverse populations or in specific subgroups such as patients with diabetes. Given these limitations, machine learning (ML) approaches have emerged as promising tools to enhance diagnostic accuracy. Artificial neural networks, in particular, can capture complex, nonlinear relationships between clinical features and disease states. Yet, few studies have evaluated their generalizability across large, heterogeneous populations or validated them using international datasets.^13^^,^^14^

The objective of this study was to develop a neural network model trained on a nationwide dataset of 20,000 individuals and to externally validate its performance using an independent international cohort. The model was compared with existing indices (FLI, HSI, ZJU, MASLD index) for the identification of MASLD, with particular attention to achieve diagnostic accuracy and applicability across demographic subgroups.

## Methods

This study was conducted in accordance with the Transparent Reporting of a Multivariable Prediction Model for Individual Prognosis or Diagnosis guidelines for transparent reporting of multivariable prediction models.^15^^,^^16^ A completed Transparent Reporting of a Multivariable Prediction Model for Individual Prognosis or Diagnosis checklist is provided as Table A5. We utilized a large retrospective cohort comprising 20,000 adult participants who underwent standardized annual health checkups at Ehime General Health Care Association between 2010 and 2020. Individuals were excluded if they had any known chronic liver disease, including serologically confirmed hepatitis B or C infection, autoimmune liver disease, or alcohol-associated liver disease (defined as alcohol consumption ≥30 g/day for men or ≥20 g/day for women). Participants with clinical or imaging-based evidence of cirrhosis were also excluded. To ensure robust model validation, the dataset was chronologically split into a development cohort of 10,000 cases (2010–2015) and an internal validation cohort of 5000 cases (2016–2020). The remaining 5000 cases were excluded due to incomplete data or ineligibility based on the criteria above.

Model development was performed using a feedforward neural network built in Keras with a TensorFlow backend. Input variables included anthropometric and clinical parameters (body mass index [BMI], abdominal circumference [AC]), as well as derived indices such as the FLI, HSI, ZJU index, and the nonalcoholic fatty liver disease index, referred to herein as the METABOLIC DYSFUNCTION–ASSOCIATED FATTY LIVER DISEASE index to reflect recent nomenclature changes. The neural network consisted of 2 fully connected hidden layers with 64 and 32 units, respectively, using rectified linear unit activation. A dropout rate of 0.3 was applied to each hidden layer to reduce overfitting. The output layer consisted of a single neuron with a sigmoid activation function to predict the probability of hepatic steatosis. The architecture of the neural network is illustrated in.

**Figure 1:**
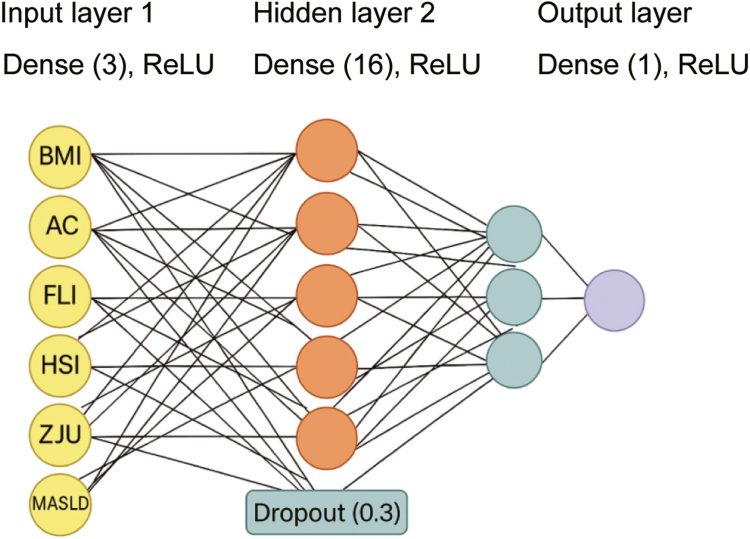
Architecture of the neural network used to predict moderate-to-severe hepatic steatosis. The model accepts six input features—BMI, AC, FLI, HSI, ZJU index, and the MASLD index It includes 2 hidden layers (Dense[64], Dense[32]) with ReLU activation and dropout (rate = 0.3), followed by a sigmoid-activated output layer for binary classification. ReLU, rectified linear unit.

Figure 1. Hyperparameters were optimized through grid search with 5-fold cross-validation in the development cohort. Model training used the Adam optimizer with a learning rate of 0.001 and binary cross-entropy as the loss function. All input variables were standardized. Missing values (<2% per variable) were imputed using multiple imputation by chained equations, incorporating all candidate predictors and the outcome variable. The imputation model included all predictor variables (BMI, AC, FLI, HSI, ZJU index, MASLD index) and the outcome variable (hepatic steatosis) to ensure appropriate handling of missing data patterns. Outliers were winsorized at the 1st and 99th percentiles.

**Figure 2:**
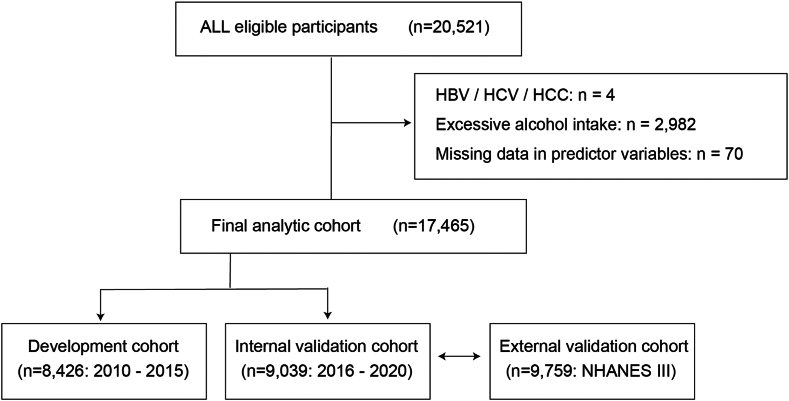
Study flowchart showing the development, internal validation, and external validation cohorts. Among 20,521 eligible participants, 3057 were excluded due to known liver disease (HBV or HCV or HCC), excessive alcohol intake (≥30 g/day for men or ≥20 g/day for women), or missing data on predictor variables. The final analytic cohort (n = 17,464) was split chronologically into a development cohort (n = 8426; 2010–2015) and an internal validation cohort (n = 9039; 2016–2020). External validation was performed using 9759 participants from the National Health and Nutrition Examination Survey III (NHANES III; 1988–1994), classified by ultrasonographic steatosis severity. HBV, hepatitis B virus; HCC, hepatocellular carcinoma; HCV, hepatitis C virus.

Internal validation was performed using the temporally independent 2016–2020 cohort. External validation was conducted using data from the Third National Health and Nutrition Examination Survey (NHANES III) cohort (1988–1994), which includes ultrasonographic assessment of hepatic steatosis not available in later cycles. In this cohort, steatosis was classified using the Gallbladder and Upper Abdominal Ultrasound Hepatic Steatosis Profile Rating variable, where scores of 2 or 3 corresponded to moderate-to-severe steatosis, and scores of 0 or 1 indicated normal-to-mild steatosis. The flow of participants and the validation strategy are summarized in Figure 2.

Model performance was evaluated using the area under the receiver operating characteristic curve (AUC). Optimal thresholds were determined using the Youden index in the development cohort, which maximizes sensitivity and specificity and is standard in diagnostic modeling, along with cutoffs prioritizing high sensitivity (≥90%) and high specificity (≥90%). Diagnostic performance was summarized by sensitivity, specificity, positive predictive value, negative predictive value, and likelihood ratios. Model calibration was assessed using the Hosmer–Lemeshow test and Brier score. Receiver operating characteristic curves and calibration plots were constructed for visual inspection. Subgroup analyses were prespecified by age (<50 vs ≥50 years), sex (male vs female), and BMI (<25 vs ≥25 kg/m^2^). Comparative performance of the neural network was benchmarked against the FLI, HSI, ZJU, and METABOLIC DYSFUNCTION–ASSOCIATED FATTY LIVER DISEASE indices across all validation settings.

All statistical analyses were conducted in R (version 4.3.1, R Foundation for Statistical Computing, Vienna, Austria), using the packages for Receiver Operating Characteristics analysis, mice, caret, and rms.

The derivation cohort was approved by the Ethics Committee of Ehime University Hospital (Approval Number: 1104005) for the analysis of health checkup data obtained from Ehime General Health Care Association. The requirement for informed consent was waived due to the retrospective and deidentified nature of the dataset. NHANES III data are publicly available through the US Centers for Disease Control and Prevention and exempt from institutional review board oversight. Code for model training and validation is available upon request to the corresponding author and subject to institutional review.

## Results

### Baseline Characteristics

The study included 8426 participants in the development cohort (2010–2015), 9039 in the internal validation cohort (2016–2020), and 9759 individuals from NHANES III for external validation. Demographic and clinical characteristics of all cohorts are summarized in Table 1. Among the 3 cohorts, the internal validation cohort had the highest mean age (54.1 ± 11.8 years), followed by the external validation cohort (53.7 ± 13.0 years) and the development cohort (52.3 ± 12.4 years). The prevalence of ultrasound-assessed hepatic steatosis was higher in the NHANES III cohort (40.1%) than in the development (32.5%) and internal validation cohorts (35.8%) (*P* < .001).

**Table 1:** Baseline Characteristics of the Development, Internal Validation, and External Validation Cohorts

Variable	Development mean (Dev)	Internal validation mean (Int)	External validation mean (Ext)	SMD (Dev vs int)	SMD (Dev vs Ext)
Age, y, mean (SD)	52.3	54.1	53.7	1.8	1.4
BMI, kg/m^2^	26.4	27	27.2	0.6	0.8
AC, cm	90.5	91.3	92.1	0.8	1.6
ALT, U/L	35.1	36.4	37.2	1.3	2.1
AST, U/L	28.7	29.5	30	0.8	1.3
GGT, U/L	45.3	47.1	46.8	1.8	1.5
FLI	49.2	51	50.6	1.8	1.4
HSI	35.6	36.2	36.8	0.6	1.2
ZJU index	22.1	22.4	22.9	0.3	0.8
MAFLD index	−0.3	−0.2	0.1	0.1	0.4

Values are presented as mean (standard deviation) unless otherwise indicated. SMDs were generally <2.0 across key variables, supporting overall comparability among cohorts.

AST, aspartate aminotransferase; GGT, gamma-glutamyltransferase; MAFLD, metabolic dysfunction–associated fatty liver disease; SD, standard deviation.

Compared with the Japanese cohorts, NHANES III participants had a higher mean BMI (27.2 ± 5.0 vs 26.4 ± 4.8 kg/m^2^; standardized mean difference (SMD) = 0.24), a larger AC (92.1 ± 11.5 vs 90.5 ± 10.8 cm; SMD = 0.15), and elevated alanine aminotransferase (ALT) levels (37.2 ± 19.4 vs 35.1 ± 18.9 U/L; SMD = 0.11). The proportion of males was comparable across cohorts (development: 58.2%, internal: 60.3%, external: 59.1%; *P* = .32).

### Model Performance

**Figure 3:**
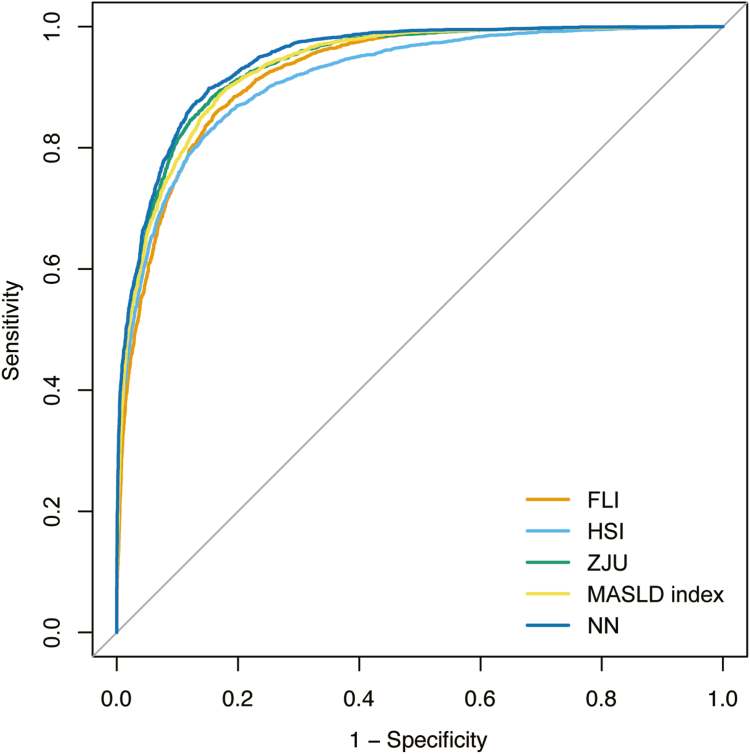
ROC curves of the neural network and conventional indices in the NHANES III external validation cohort. The neural network achieved the highest AUC (= 0.924), significantly outperforming conventional indices, including FLI (0.842), HSI (0.834), ZJU (0.856), and the MASLD index (0.867) (all *P* < .001 by DeLong test). NN, neural network.

The neural network achieved robust discrimination in both the internal validation cohort (AUC = 0.922, 95% confidence interval (CI): 0.913–0.931) and the external validation cohort (AUC = 0.924, 95% CI: 0.917–0.931), outperforming all conventional indices (Figure 3). In the external validation cohort of 9759 individuals, calibration metrics included a slope of 1.032 (95% CI: 1.002–1.062) and intercept of −0.122 (95% CI: −0.144 to −0.099). In the NHANES III external validation dataset, the AUCs of conventional indices were as follows: FLI, 0.842; HSI, 0.834; ZJU index, 0.856; and MASLD index, 0.867. The superiority of the neural network over all conventional indices was statistically significant (*P* < .001 for all pairwise comparisons using DeLong’s test; Table A1).

At the threshold maximizing the Youden Index (cutoff = 0.357), the neural network demonstrated a sensitivity of 0.89 and a specificity of 0.86. When the cutoff was adjusted to ensure a fixed sensitivity of 90%, the resulting specificity was 0.74. Conversely, at the cutoff achieving a fixed specificity of 90%, the resulting sensitivity was 0.74. Across all 3 thresholding strategies, the neural network model consistently outperformed the FLI, HSI, ZJU, and MASLD indices in diagnostic accuracy (Table A1).

To address concerns regarding patients with normal liver function tests (LFTs), we conducted a subgroup analysis restricted to individuals with aspartate aminotransferase ≤30 IU/L, ALT ≤30 IU/L, and gamma-glutamyltransferase ≤50 IU/L (n = 15,034). In this biochemically normal subgroup, the model demonstrated excellent discrimination (AUC = 0.887), suggesting robust performance even among patients where steatosis detection is most challenging. Calibration intercept and slope were −2.183 (95% CI: −2.244 to −2.122) and 1.241 (95% CI: 1.194−1.288), respectively. These results are provided in Table A6.

**Figure 4:**
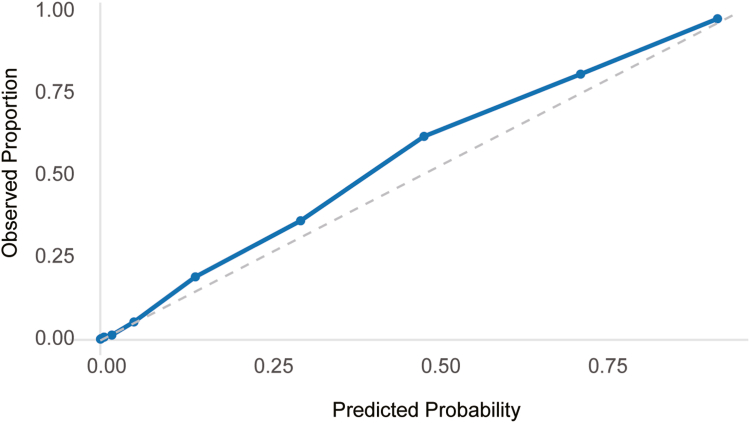
Calibration curve for the neural network model in the NHANES III external validation cohort. Predicted probabilities were divided into deciles. The observed event rate (the proportion of individuals with ultrasound-defined moderate-to-severe hepatic steatosis) closely matched the predicted risk, demonstrating good calibration across the full range. Hosmer–Lemeshow test: *P* = .31.

Calibration performance was acceptable in the external validation cohort, as indicated by the Hosmer–Lemeshow test (*P* = .31). The calibration curve (Figure 4) demonstrated close agreement between predicted and observed probabilities across deciles of risk, supporting the reliability of the model's probabilistic output. The neural network yielded the lowest Brier score (0.116, 95% CI: 0.111–0.121), significantly better than the FLI (0.128), HSI (0.133), ZJU (0.136), and MASLD index (0.141) (all *P* < .001).

**Figure 5:**
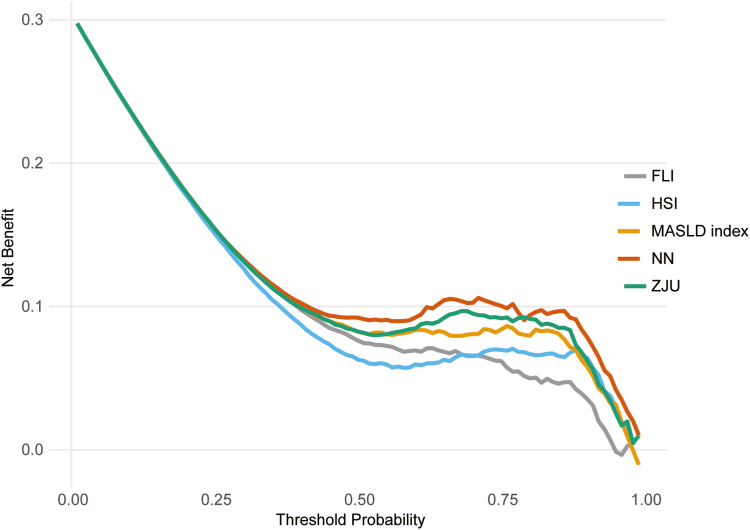
Decision curve analysis for moderate-to-severe hepatic steatosis in the external validation cohort. Across a range of threshold probabilities (10%–90%), the neural network consistently showed a greater net clinical benefit than conventional indices (FLI, HSI, ZJU, and MASLD index), as evaluated using decision curve analysis. NN, neural network.

Feature importance analysis using permutation-based methods indicated that BMI was the most influential predictor (28.3%), followed by AC (23.7%) and FLI (19.2%). These results suggest that anthropometric measures had the greatest impact on model predictions (Figure A1). Figure A2 provides an individual-level visualization of these effects, illustrating how higher values of BMI, AC, and FLI are associated with an increased predicted risk. Decision curve analysis demonstrated a consistently higher net benefit for the neural network over conventional indices across a wide range of threshold probabilities (10%–90%) (Figure 5).

### Subgroup Analysis

**Figure 6:**
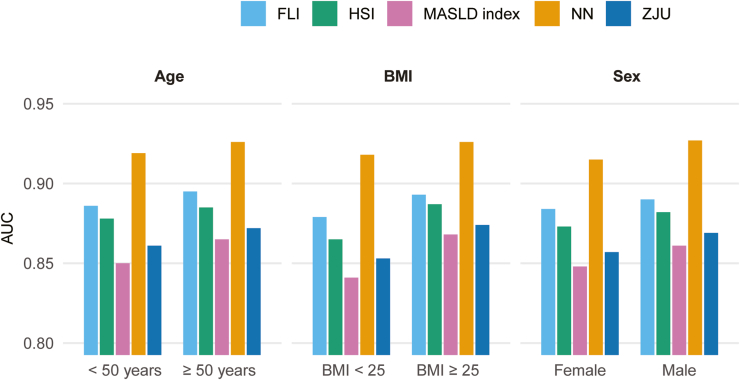
Subgroup analysis of AUC performance for the neural network and conventional indices in the NHANES III cohort. The neural network maintained high discrimination across all subgroups: sex (male AUC = 0.927, female AUC = 0.915; interaction *P* = .14), age (<50 years = 0.919, ≥50 years = 0.926; *P* = .38), and BMI strata (<25 kg/m^2^ = 0.918, ≥25 kg/m^2^ = 0.926; *P* = .42), with no significant interactions observed. NN, neural network.

Subgroup analysis confirmed consistent model performance across all strata (Figure 6, Table A2). In NHANES III, AUCs were 0.927 (95% CI: 0.917–0.937) for males and 0.915 (95% CI: 0.903–0.927) for females (interaction *P* = .14). By age, AUCs were 0.919 (95% CI: 0.907–0.931) in participants <50 years and 0.926 (95% CI: 0.916–0.936) in those ≥50 years (interaction *P* = .38). By BMI, AUCs were 0.918 (95% CI: 0.903–0.933) for BMI <25 kg/m^2^ and 0.926 (95% CI: 0.917–0.935) for BMI ≥25 kg/m^2^ (interaction *P* = .42).

Across all subgroups, the neural network consistently outperformed conventional indices, with relative AUC gains ranging from 3.4% to 9.2% (all *P* < .01). These findings remained consistent across different thresholding strategies (sensitivity- or specificity-focused), as detailed in Tables A3 and A4.

## Discussion

In this large-scale externally validated study, we demonstrated that a neural network model trained on 20,000 Japanese health checkup participants significantly outperformed 4 widely used noninvasive indices—FLI, HSI, ZJU, and MASLD index—in identifying moderate-to-severe hepatic steatosis in an independent US population. The model exhibited strong discriminatory ability across demographic and clinical subgroups, was well-calibrated, and showed higher clinical utility as evidenced by decision curve analysis. These findings support the potential role of artificial intelligence–driven approaches in the early identification of MASLD.

Our results expand on previous comparative studies of conventional indices. FLI and HSI have shown moderate diagnostic accuracy in diverse populations (AUCs 0.83–0.85)^9^^,^^10^, yet their performance is often compromised in lean individuals and ethnically heterogeneous cohorts.^17^^,^^18^ The superior AUC of 0.924 for our neural network likely reflects its ability to model nonlinear and higher-order interactions among clinical parameters that traditional regression-based tools cannot capture.^19^^,^^20^ This was especially evident in subgroups such as normal-weight individuals (AUC = 0.918), where conventional indices typically underperform.^21^

The clinical imperative for early MASLD detection is underscored in recent guidelines from European Association for the Study of the Liver,^5^ American Association for the Study of Liver Diseases,^6^ and Japan Society of Hepatology,^7^ all of which recommend screening high-risk individuals. However, reliance on ultrasound as the first-line modality poses challenges: limited availability, operator dependency, and reduced accuracy in obese populations.^22^ The model we propose may help overcome these barriers by functioning as a preimaging triage tool, facilitating risk stratification based on standard clinical data. The model aims to enhance early detection and referral for imaging-based confirmation, minimizing unnecessary interventions.

This study offers several methodological strengths. First, time-split internal validation minimized temporal bias, an often overlooked but critical factor in ML research.^23^ Second, external validation in NHANES III—a large, ethnically diverse, population-based dataset—supports the generalizability of our findings. Third, our use of comprehensive evaluation metrics, including receiver operating characteristic analysis, calibration, and decision curves, provides a multifaceted assessment of performance. Finally, interpretability was addressed through permutation-based feature importance, identifying AC and BMI as the 2 most influential variables.

The dominance of AC over BMI aligns with growing evidence that central adiposity may better predict hepatic steatosis and metabolic dysfunction than overall obesity.^24^, ^25^, ^26^ Laboratory parameters such as ALT and TG, although individually weak predictors, contributed meaningfully in aggregate, highlighting the value of neural networks in integrating multivariate signals to generate more precise predictions.

In addition, while our sex-stratified analysis revealed no statistically significant interaction (AUC: 0.927 in males vs 0.915 in females; interaction *P* = .14), we acknowledge that biological sex may influence hepatic fat accumulation, metabolic profiles, and diagnostic performance. Although our unified model performed well across sexes, future studies could consider sex-stratified modeling or explore sex-specific disease mechanisms. This approach is in accordance with the SAGER guidelines for transparent reporting of sex and gender in research.

Some limitations must be acknowledged. The reference standard for hepatic steatosis was ultrasonography, which, although widely used, is less accurate than liver biopsy or magnetic resonance imaging-proton density fat fraction.^27^, ^28^, ^29^ Second, the NHANES III dataset was collected in the late 20th century and may not fully reflect contemporary disease patterns. Third, the model was not designed to distinguish steatosis from more advanced disease states such as steatohepatitis or fibrosis. Fourth, comparative evaluation against other ML architectures (eg, gradient boosting, ensemble models) or emerging biomarkers (e.g., FAST, FibroScan-CAP) was not performed. Finally, external validation in prospective, contemporary clinical settings remains a necessary next step.

Future directions include (1) prospective validation in diverse clinical populations, (2) integration with fibrosis and inflammation markers for comprehensive MASLD staging, (3) development of simplified point-based tools derived from the neural network for low-resource settings, and (4) exploration of longitudinal predictive value for clinical outcomes such as fibrosis progression, cardiovascular events, or mortality.

## Conclusion

Our findings suggest that neural network models can substantially improve the noninvasive detection of hepaticsteatosis in MASLD. The model demonstrated robust performance, calibration, and clinical utility across population subgroups. Its implementation in clinical workflows may enable earlier detection and intervention, ultimately helping to mitigate the rising global burden of MASLD.

## Figures and Tables

**Supplementary Figure S1:**
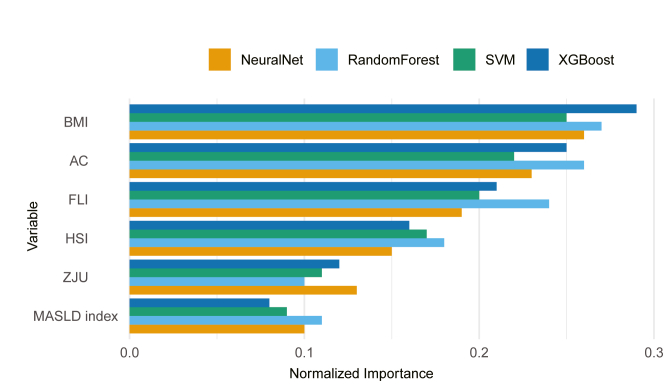


**Supplementary Figure S2:**