# Supplementary material for: Development of a Neural Network to Detect Hepatic Steatosis in Metabolic Dysfunction–Associated Steatotic Liver Disease
Source: Gastro Hep Adv. 2025 Aug 19;5(1):100765. doi: 10.1016/j.gastha.2025.100765 (PMC12546783; doi:10.1016/j.gastha.2025.100765)
Supplement: Supplemental Material [file mmc1.docx]

**Supplemental Table S1.** **Diagnostic performance of the neural network and conventional non-invasive indices (FLI, HSI, ZJU, and MAFLD index) for detecting moderate-to-severe hepatic steatosis in the NHANES III external validation cohort.**

| Index | AUC | Optimal Cutoff | Sensitivity (Optimal) | Specificity (Optimal) | Sensitivity 90% Cutoff | Specificity at  Sens 90% | Specificity 90% Cutoff | Sensitivity at Spec 90% |
| --- | --- | --- | --- | --- | --- | --- | --- | --- |
| FLI | 0.842 | 58.2 | 0.79 | 0.76 | 42.1 | 0.58 | 72.5 | 0.55 |
| HIS | 0.834 | 36.5 | 0.77 | 0.75 | 32.4 | 0.56 | 41.3 | 0.53 |
| ZJU | 0.856 | 23.1 | 0.81 | 0.78 | 19.2 | 0.61 | 27.6 | 0.59 |
| MAFLD index | 0.867 | -0.1 | 0.82 | 0.79 | -0.8 | 0.62 | 0.6 | 0.61 |
| Neural Network | 0.924 | 0.357 | 0.89 | 0.86 | 0.228 | 0.74 | 0.484 | 0.74 |

The optimal cutoff was determined using the Youden Index. Additional thresholds were selected to achieve fixed sensitivity (90%) or specificity (90%). Sensitivity and specificity are expressed as proportions.
AUC, area under the receiver operating characteristic curve; FLI, Fatty Liver Index; HSI, Hepatic Steatosis Index; MAFLD, metabolic dysfunction-associated fatty liver disease; SENS, sensitivity; SPEC, specificity; ZJU, Zhejiang University index.

**Supplemental Table S2. Subgroup analysis of area under the receiver operating characteristic curve for the neural network and conventional indices in the NHANES III external validation cohort.**

| Subgroup | NN | FLI | HSI | ZJU | MAFLD index |
| --- | --- | --- | --- | --- | --- |
| Male | 0.927 | 0.89 | 0.882 | 0.869 | 0.861 |
| Female | 0.915 | 0.884 | 0.873 | 0.857 | 0.848 |
| <50 years | 0.919 | 0.886 | 0.878 | 0.861 | 0.85 |
| ≥50 years | 0.926 | 0.895 | 0.885 | 0.872 | 0.865 |
| BMI <25 | 0.918 | 0.879 | 0.865 | 0.853 | 0.841 |
| BMI ≥25 | 0.926 | 0.893 | 0.887 | 0.874 | 0.868 |

NN, Neural network; FLI, Fatty Liver Index; HSI, Hepatic Steatosis Index; ZJU, Zhejiang University index; MAFLD, metabolic dysfunction-associated fatty liver disease

**Supplemental Table S3. Relative improvement in AUC (%) of the neural network compared to conventional indices across all subgroups in the NHANES III cohort.**

| Subgroup | FLI | HSI | ZJU | MAFLD |
| --- | --- | --- | --- | --- |
| Male | 4.2 | 5.1 | 6.7 | 7.7 |
| Female | 3.5 | 4.8 | 6.8 | 7.9 |
| <50 years | 3.7 | 4.7 | 6.7 | 8.1 |
| ≥50 years | 3.5 | 4.6 | 6.2 | 7.1 |
| BMI <25 | 4.4 | 6.1 | 7.6 | 9.2 |
| BMI ≥25 | 3.7 | 4.4 | 5.9 | 6.7 |

FLI, Fatty Liver Index; HSI, Hepatic Steatosis Index; ZJU, Zhejiang University index; MAFLD, metabolic dysfunction-associated fatty liver disease

**Supplemental Table S4. Diagnostic accuracy of the neural network (NN) and fatty liver index (FLI) at clinically relevant thresholds.**

| Subgroup | NN  Spec (Sens ≥90%) | FLI  Spec (Sens ≥90%) | NN  Sens (Spec ≥90%) | FLI  Sens (Spec ≥90%) |
| --- | --- | --- | --- | --- |
| Male | 0.746 | 0.661 | 0.731 | 0.608 |
| Female | 0.738 | 0.65 | 0.718 | 0.596 |
| <50 years | 0.721 | 0.637 | 0.709 | 0.587 |
| ≥50 years | 0.754 | 0.666 | 0.738 | 0.616 |
| BMI <25 | 0.734 | 0.628 | 0.721 | 0.589 |
| BMI ≥25 | 0.752 | 0.665 | 0.736 | 0.613 |

Sensitivity-focused thresholds were set to achieve ≥90% sensitivity, and specificity-focused thresholds to achieve ≥90% specificity.
Data are presented by sex, age, and BMI strata in the NHANES III cohort.
NN, neural network; FLI, Fatty Liver Index

**Supplementary Table S5. TRIPOD Checklist for Reporting of Prediction Model Development and Validation**

| **Section/Topic** | **Item** | **Checklist Item** | **Page No.** |
| --- | --- | --- | --- |
| Title and Abstract | 1 | Title and structured abstract included | 1, 6-8 |
| Introduction | 2 | Background and objectives clearly stated | 12-14 |
| Methods: Source of data | 3 | Study design, data sources, inclusion and exclusion criteria specified | 14-15 |
| Participants | 4 | Eligibility criteria, participant flow described | 14-15 |
| Outcome | 5 | Definition of hepatic steatosis described | 15, 17 |
| Predictors | 6 | Predictor variables and definitions described | 15, 16 |
| Sample size | 7 | Sample size and handling of missing data explained | 14-15 |
| Missing data | 8 | Imputation details fully reported | 16 |
| Statistical analysis methods | 9 | Model development and validation methods described | 15-18 |
| Risk groups | 10 | No risk groupings used (not applicable) | N/A |
| Results: Participants | 11 | Flow diagram and baseline characteristics provided | 19 |
| Model development | 12 | Model specification, predictor selection described | 15-16 |
| Model performance | 13 | Performance measures (AUC, calibration, Brier score) reported | 20-23 |
| Results validation | 14 | Internal and external validation results presented | 20-23 |
| Discussion: Limitations | 15 | Study limitations discussed | 28 |
| Interpretation | 16 | Interpretation of results discussed in clinical context | 24-29 |
| Implications | 17 | Potential clinical applications discussed | 28-29 |
| Supplementary information | 18 | Supplementary Tables S5-S6 provided | Suppl. |
| Funding | 19 | Funding and conflicts of interest disclosed | 2 |

**Supplementary Table S6. Model Performance in Normal LFT Subgroup**

| **Metric** | **Estimate** | **95% CI** |
| --- | --- | --- |
| Sample Size (n) | 15,034 | — |
| AUC | 0.887 | — |
| Brier Score | 0.186 | — |
| Calibration Intercept | -2.183 | -2.244 to -2.122 |
| Calibration Slope | 1.241 | 1.194 to 1.288 |
